# Clinical outcomes after anterior cruciate ligament injury: panther symposium ACL injury clinical outcomes consensus group

**DOI:** 10.1007/s00167-020-06061-x

**Published:** 2020-08-06

**Authors:** Eleonor Svantesson, Eric Hamrin Senorski, Kate E. Webster, Jón Karlsson, Theresa Diermeier, Benjamin B. Rothrauff, Sean J. Meredith, Thomas Rauer, James J. Irrgang, Kurt P. Spindler, C. Benjamin Ma, Volker Musahl, The Panther Symposium ACL Injury Clinical Outcomes Consensus Group, Freddie H. Fu, Olufemi R. Ayeni, Francesco Della Villa, Stefano Della Villa, Scott Dye, Mario Ferretti, Alan Getgood, Timo Järvelä, Christopher C Kaeding, Ryosuke Kuroda, Bryson Lesniak, Robert G. Marx, Gregory B Maletis, Leo Pinczewski, Anil Ranawat, Bruce Reider, Romain Seil, Carola van Eck, Brian R Wolf, Patrick Yung, Stefano Zaffagnini, Minghao Zheng

**Affiliations:** 1 Department of Orthopaedics, Institute of Clinical Sciences, Sahlgrenska Academy, University of Gothenburg, Gothenburg, Sweden; 2 Gothenburg Sports and Trauma Research Center, Gothenburg, Sweden; 3 Department of Rehabilitation and Health, Institute of Neuroscience and Physiology, Sahlgrenska Academy, University of Gothenburg, Gothenburg, Sweden; 4 School of Allied Health, La Trobe University, Melbourne, VIC Australia; 5 Department of Orthopaedics, Sahlgrenska University Hospital, Mölndal, Sweden; 6 Department of Sportorthopedics, Klinikum rechts der Isar Technische Universitat Munchen, Munich, Germany; 7 UPMC Freddie Fu Sports Medicine Center, Department of Orthopaedic Surgery, University of Pittsburgh, 3200 S Water St, Pittsburgh, 15203 PA USA; 8 Department of Orthopaedics, University of Maryland School of Medicine, Baltimore, MD USA; 9 Department of Trauma Surgery, University Hospital Zurich, Zurich, Switzerland; 10 Department of Orthopaedic Surgery, University of Pittsburgh Medical Center, Pittsburgh, PA USA; 11 Department of Physical Therapy, University of Pittsburgh, Pittsburgh, PA USA; 12 Cleveland Clinic Sports Health Center, Garfield Heights, OH USA; 13 Department of Orthopedic Surgery, University of California, San Francisco, California USA

**Keywords:** ACL, Anterior cruciate ligament, Outcome, Consensus statement, Reconstruction

## Abstract

**Purpose:**

A stringent outcome assessment is a key aspect for establishing evidence-based clinical guidelines for anterior cruciate ligament (ACL) injury treatment. The aim of this consensus statement was to establish what data should be reported when conducting an ACL outcome study, what specific outcome measurements should be used and at what follow-up time those outcomes should be assessed.

**Methods:**

To establish a standardized approach to assessment of clinical outcome after ACL treatment, a consensus meeting including a multidisciplinary group of ACL experts was held at the ACL Consensus Meeting Panther Symposium, Pittsburgh, PA; USA, in June 2019. The group reached consensus on nine statements by using a modified Delphi method.

**Results:**

In general, outcomes after ACL treatment can be divided into four robust categories—early adverse events, patient-reported outcomes, ACL graft failure/recurrent ligament disruption and clinical measures of knee function and structure. A comprehensive assessment following ACL treatment should aim to provide a complete overview of the treatment result, optimally including the various aspects of outcome categories. For most research questions, a minimum follow-up of 2 years with an optimal follow-up rate of 80% is necessary to achieve a comprehensive assessment. This should include clinical examination, any sustained re-injuries, validated knee-specific PROs and Health-Related Quality of Life questionnaires. In the mid- to long-term follow-up, the presence of osteoarthritis should be evaluated.

**Conclusion:**

This consensus paper provides practical guidelines for how the aforementioned entities of outcomes should be reported and suggests the preferred tools for a reliable and valid assessment of outcome after ACL treatment.

**Level of evidence:**

V.

**Electronic supplementary material:**

The online version of this article (10.1007/s00167-020-06061-x) contains supplementary material, which is available to authorized users.

## Introduction

The evolution of evidence-based medicine is considered as one of the most important paradigm shifts in modern medicine [29, 105], for which conduction of high-quality research is fundamental. Anterior cruciate ligament (ACL) injuries are among the most studied in the field of orthopedics and sports medicine, with over 25,000 publications available in the PubMed database up to mid-2019. Despite ongoing research and advancements in treatment regimens for ACL injuries over the past decades, the goal of restored knee function and preserved long-term knee-related health remains a challenge. Re-injury rates are high, especially among the young and active [115, 119], and the high rate of subsequent development of post-traumatic osteoarthritis (OA) is worrying [1, 22, 82, 87]. In the best interest of our patients, a deepened understanding of how to optimize an individualized approach to ACL injury treatment is needed. One important part of this process is to strive for a standardized and homogenous research methodology of clinical outcome assessment after ACL treatment.

A rigorous outcome assessment after ACL injury is a key aspect for determining the clinical efficacy and effectiveness of treatment. It can also identify modifiable and non-modifiable predictors of good and poor outcome, which provide valuable insights for the patient’s prognosis and should be discussed in the context of shared decision-making for the treatment choice after ACL injury. Moreover, a standardized outcome assessment and reporting of data is required for comparisons between studies and for pooling of data in meta-analyses to provide the highest level of evidence-based medicine. Current literature related to ACL treatment is limited by the fact that no consensus exists on how to assess and report clinical outcome. There is a wide range of validated outcomes assessment tools for ACL treatment. Although each of these outcome measures may offer certain advantages and the patient’s perspective of outcome should always be evaluated, caution must be taken to ensure that outcome measures accurately capture patient-centered and clinically relevant outcomes for an ACL injured patient. Another debated area in ACL outcome assessment is the use of “ACL graft failure” as an endpoint for research. This is highly relevant to the patient, however, there is no universally accepted definition of graft failure utilized in the literature. Moreover, the lack of a consistent approach as to the timing of when outcomes should be measured following treatment and how such measures are reported makes an appraisal of the current literature challenging, which limits the recommendations for the patient’s best possible care.

As the body of evidence on ACL treatment grows, there is an urgent need to reach consensus on how clinical outcome should be assessed and reported. Surgeons and researchers should strive to create optimal conditions for appraisal of the cumulative evidence regarding ACL treatment, thereby promoting an evidence-based approach by using outcome measures that are reliable, valid, responsive over time and comparable. Therefore, a multidisciplinary group of experts was assembled for an international consensus meeting aiming to establish a standardized approach to clinical outcome assessment for patients receiving ACL treatment, i.e. both operative and non-operative treatment [80]. The purpose of this article is to provide the results from the consensus meeting in terms of what outcomes should be reported when conducting an ACL outcome study, the recommended outcome measurements, and at which follow-up time points those measurements should be used.

## Materials and methods

A multidisciplinary panel of national and international experts in ACL injury, including orthopedic surgeons, physical medicine and rehabilitation physicians, physical therapists, and scientists, were convened in a 1-year consensus-building effort, which culminated in the ACL Consensus Meeting Panther Symposium held at the University of Pittsburgh and University of Pittsburgh Medical Center in Pittsburgh, PA, USA, in June 2019. The symposium included delegates from 18 countries encompassing 6 continents. The working group of this topic consisted of 25 participants.

A list of 13 statements on clinical outcomes was drafted by the steering committee of the meeting based on current literature and controversies in clinical outcome assessment. The consensus group members completed an online survey addressing the 13 statements prior to the ACL consensus meeting. The initial statements and corresponding responses are found in the supplementary material (Online Appendix 1).

A modified Delphi consensus discussion for each of the 13 statements was subsequently held at the in-person consensus meeting. The session was moderated by two senior researchers (KW and JK). Each statement was discussed and revised by the working group, after which a vote on an agreement with the statement was performed. No count was held on the number of roundtables, but the discussion was continued until consensus was met for each statement. A majority of 80% agreement was determined a priori as being a satisfactory level of consensus. Opposing views were documented and it was determined that those statements for which 80% agreement was not achieved should be discussed in the paper, noting the percentage of agreement and accompanied with the discussion held during the meeting. Statements that the panel determined as irrelevant, redundant or overlapping with another statement were either excluded or combined with the overlapping statement. Statement 2 in this consensus paper was combined from two original statements (originally statement 10 and 11 in the online survey, Online Appendix 1) because these were considered as overlapping. There was 100% agreement for the original statement 10, and when proceeding to discussion and voting on the original statement 11, the panel instead agreed to combine statement 10 and 11 into one. However, no formal voting was undertaken for the finalized combination of the two. Thus, the percentage of agreement for statement 2 in this consensus paper could not be reported.

**Fig. 1:**
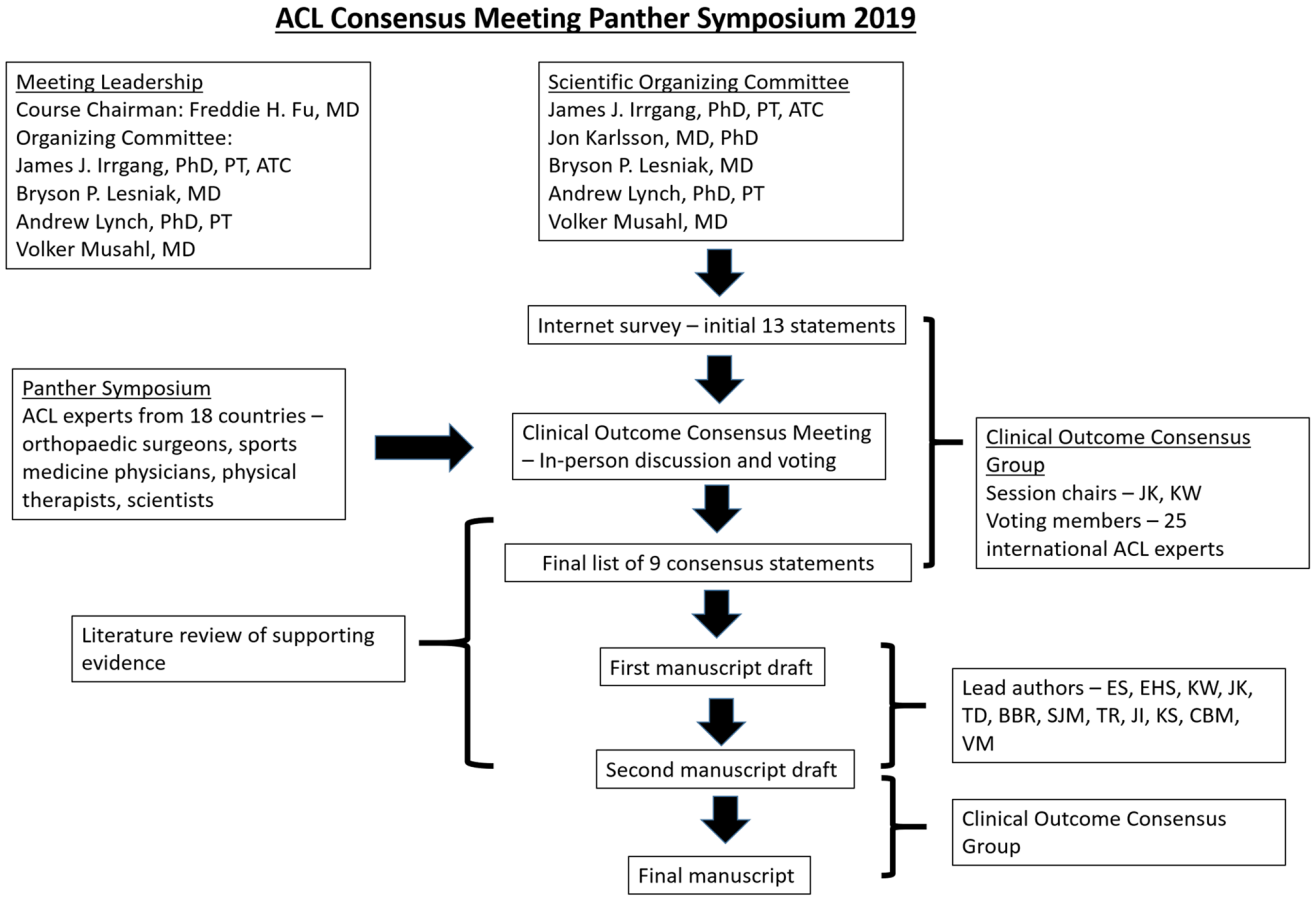
The process of the consensus project

This working group was assigned two liaisons (ES and EHS) who were responsible for amending each statement as requested over the course of the discussion. Liaisons transcribed the discussion, performed data analyses, and subsequently completed a MEDLINE literature review for each finalized statement. To reduce the potential for bias in the data analysis and/or literature review, liaisons did not submit answers to the online questionnaire, nor did they partake in the voting process. A description of the consensus process is presented in Fig. 1 and a list of definitions used at the consensus meeting for the specific statements is provided in Table 1.

**Table 1:** Operational definitions

Chronic ACL injury	A non-operatively treated ACL injury with persistent complaints of instability more than 6 months after the initial injury
Acute ACL reconstruction	An ACL reconstruction taking place within 3 months from injury
Delayed ACL reconstruction	An ACL injury that is planned to be treated with reconstruction and take place after 6 months from injury, or an ACL reconstruction that takes place after non-operative treatment has been tried without a satisfactory outcome
Instability	A patient’s perception of the knee not feeling stable
Laxity	The passive displacement of the knee joint when an external force or torque is applied

*ACL* anterior cruciate ligament

## Consensus statements and discussion

Of the 13 statements discussed by the working group, 9 achieved consensus, and 4 were excluded since these were considered to include information similar to one or more of the other statements. Thus, some of the nine statements achieving consensus were slightly modified to include aspects from the four excluded statements. The 9 final statements, with supporting literature review, are presented below. These statements are presented in three main sections for readability purposes; (1) Planning for outcome assessment (2) Clinical outcome assessment (3) Patient-reported outcome. An overview of the consensus statements is presented in Table 2.

**Table 2:** Summary of the consensus statements for clinical outcome assessment after ACL injury

**Planning for outcome assessment**
1. A priori power calculation of sample size in relation to the primary endpoint must be performed and reported to avoid under-powered studies 2. Improvement from pre-treatment status is the outcome of interest. Minimum description of pre-treatment status should include demographic data, validated knee-specific PRO assessment, HRQoL and measure of type and level of pre-injury sport/activity
**Clinical outcome assessment**
3. Minimal length of follow-up when reporting outcomes depends on the outcome being assessed and should optimally include 80% of the entire cohort 4. Comprehensive assessment after ACL surgery (minimum 2 years) should include adverse events, clinical measures of knee function and structure, PRO, activity level, and recurrent ligament disruption 5. Comprehensive assessment after ACL surgery in the medium to long-term (5 + years) should also include measures of post-traumatic osteoarthritis 6. Clinical assessment of ACL injury treatment should include measures of anteroposterior and rotatory knee laxity
**Patient-reported outcome**
7. Assessment of PRO should optimally include at least one knee-specific outcome tool, one activity rating scale, and one measure of health-related quality of life 8. The IKDC Subjective Knee Form is the recommended knee-related outcome measure for ACL injury and treatment 9. Measurement of the patient acceptable symptom state (PASS) is valuable in the assessment of the outcome of ACL injury and treatment

*ACL* anterior cruciate ligament, *HRQoL* Health-related quality of life, *IKDC* International Knee Documentation Committee, *PRO* patient-reported outcome

## Planning for outcome assessment

### A priori power calculation of sample size in relation to the primary end point must be performed and reported to avoid under-powered studies

(25/25, 100% agreement)

“Sample size is key to avoiding underpowered studies—We should always try to perform high-quality research, and power calculation is part of this.”

A critical point when evaluating a study outcome is to ensure that the sample size is large enough to detect a difference when a true difference in fact exists. Otherwise, the study may be under-powered and subject to Beta error (Type II error). This can have serious consequences on clinical practice if no difference in outcome is concluded to exist between for example, two interventions even though one of the interventions is truly inferior, or superior, compared with the other. Ultimately, under-powered studies fail to identify the best possible care for our patients. Approximately two-thirds of randomized controlled trials related to ACL reconstruction failed to report an a priori sample size calculation [5, 94]. Although a more recent assessment of the literature shows that these numbers have substantially improved since 2009 [55], improvements can be made. A study should have a power of at least 80% (1 − *β*), which means that the risk of a Type II error, or false negative result, is 20%. A priori power calculation helps to ensure that the sample size will be large enough to minimize the risk of Type II error. The power calculation should be determined for the primary patient-centered endpoint, meaning that if an endpoint is chosen that has a low event rate, the study sample size will need to be larger than if one expects that many patients will reach the endpoint. The sample size calculation, therefore, aids in the determination of feasibility and will help reduce the rate of incomplete studies and wasted resources. It is also an ethical responsibility to perform a sample size calculation since it is unethical to include substantially more patients than necessary. In relation to large registry studies, a power calculation may be redundant, but this can depend on the outcome. It is therefore recommended that a statement on power always should be included. A sample size calculation should be performed whenever possible prior to the start of the study. However, a post hoc power calculation to test the validity of the study results can be an acceptable method under certain conditions, for instance in the case of a retrospective study, but caution must be given to the high risk of overestimating power [39, 113].

*Conclusion* Researchers must report the power of the study to ensure that the sample size is sufficient to detect a difference if one truly existed and to give readers of the paper an understanding of the strength and generalizability of the results.

### Improvement from pre-treatment status is the outcome of interest. Minimum description of pre-treatment status should include demographic data, validated knee-specific PRO assessment, HRQoL and measure of type and level of pre-injury sport/activity

“We must know where we started to determine whether the treatment was effective.”

The goal of all available treatments for an ACL injury is to improve the outcome from the pre-treatment status. Hence, without assessment of the pre-treatment status, the relative improvement cannot be measured and reported. Assessment of the pre-treatment status is also important to identify baseline variables that may confound or explain a given study result. When comparative trials are conducted, variables known to influence the outcome of interest should be equally distributed between the groups or otherwise adjusted for by using appropriate statistical methods. Adjustments can be planned a priori based on previous studies or assessed by adjusting for variables that correlate with both the predictor and outcome. Researchers should thoroughly plan data collection prior to the study start while considering their study population and their research question.

The demographic data should give an overview of the characteristics of the investigated population, which aids to determine the generalizability of the study results. Demographic data should at a minimum include patient sex, age, anthropometric data, relevant medical history and prior knee joint injuries. Family history of ACL injuries may also be relevant since a heritable component of ACL injuries appears to exist [19, 116]. Moreover, the type and level of pre-injury sport or activity should be reported to determine whether the treatment successfully returned the patients to their pre-injury activity level. The recommended tool for sport and activity assessment is the Marx activity scale [67], which has been validated and has high reliability. The Marx activity scale enables an evaluation of both the type of activity and the exposure time, which are both crucial aspects when reporting on activity. In this aspect, it differs from other measures of activity, e.g. the Tegner activity scale [106] which enables grading of activity level but does not account for activity exposure. Other validated tools for activity include, for example, the International Knee Documentation Committee Subjective Knee Form (IKDC-SKF) [51] which includes one item (item 8) related to the activity level which the patient performs on a regular basis. The item is answered by choosing one out of five responses ranging from very strenuous activity to unable to perform light activities. Classification of activity and sports participation can also be rated according to Level I-IV activity, which was included in the original version of the IKDC Knee Ligament Standard Evaluation Form [46] and is still frequently used in ACL research [31, 40, 71]. Another example of a tool for activity assessment is the Cincinnati sports activity scale [9]. The tools for activity assessment are presented in Table 3. It is of importance to further distinguish between pre-injury and pre-surgery activity level. Since a pre-surgery activity level has a risk of being representative of the patient’s activity while injured, pre-injury activity should always be reported.

**Table 3:** Tools for activity assessment

Assessment tool	Description
IKDC-SKF [51]	4-Very strenuous activities like jumping or pivoting as in basketball or soccer
	3-Strenuous activities like heavy physical work, skiing or tennis
	2-Moderate activities like moderate physical work, running or jogging
	1-Light activities like walking, housework or yard work
	0-Unable to perform any of the above activities due to knee
Tegner Activity Scale [106]	Level 10 Competitive sports- soccer, football, rugby (national elite)
	Level 9 Competitive sports- soccer, football, rugby (lower divisions), ice hockey, wrestling, gymnastics, basketball
	Level 8 Competitive sports- racquetball or bandy, squash or badminton, track and field athletics (jumping, etc.), down-hill skiing
	Level 7 Competitive sports- tennis, running, motorcars speedway, handball, Recreational sports- soccer, football, rugby, bandy, ice hockey, basketball, squash, racquetball, running
	Level 6 Recreational sports- tennis and badminton, handball, racquetball, down-hill skiing, jogging at least 5 times per week
	Level 5 Work- heavy labor (construction, etc.) Competitive sports- cycling, cross-country skiing, Recreational sports- jogging on uneven ground at least twice weekly
	Level 4 Work- moderately heavy labor (e.g. truck driving, etc.)
	Level 3 Work- light labor (nursing, etc.)
	Level 2 Work- light labor Walking on uneven ground possible, but impossible to backpack or hike
	Level 1 Work- sedentary (secretarial, etc.)
	Level 0 Sick leave or disability pension because of knee problems
Marx Activity Rating Scale [67]	Patient is asked how often the activities running, cutting, deceleration and pivoting have been performed during the last year in your healthiest and most active state. Each activity is scored on a 0-4 scale as follows:
	0- Less than one time in a month
	1-One time in a month
	2- One time in a week
	3-Two to three times in a week
	4-Four or more times in a week
Cincinnati Sports Activity Scale [9]	Divided into four major levels, with subcategories.
	Level I (participates 4–7 days/week)
	Level II (participates 1–3 days/week)
	Level III (participates 1–3 times/month)
	Level IV (no sports)
	Subcategories for level I-III (5 points decline for every step downwards, starting from 100p):
	Jumping, hard pivoting, cutting (basketball, volleyball, football, gymnastics, soccer)
	Running, twisting, turning (tennis, racquetball, handball, ice hockey, field hockey, skiing, wrestling
	No running, twisting jumping (cycling, swimming)
	Level IV with the following subcategories and points for each:
	40- Activities of daily living without problems
	20- Moderate problems with activities of daily living
	0- Severe problems with activities of daily living; on crutches, full disability
IKDC Knee Ligament Standard Evaluation form [46]	Level I- jumping, pivoting, hard cutting, football, soccer
	Level II- heavy manual work, skiing, tennis
	Level III- light manual work, jogging, running
	Level IV- activities of daily living, sedentary work

*IKDC* International Knee Documentation Committee, *IKDC-SKF* International Knee Documentation Committee Subjective Knee Form

Pre-treatment assessment of PROs is particularly valuable for patients with chronic ACL injuries, or as a pre-surgical treatment baseline for patients undergoing delayed ACL reconstruction. This is because patients with chronic ACL injury may have had the time to live with and try to cope with the potential limitations of their ACL deficient status, as opposed to the acutely injured patients who are impaired due to injury-related factors (e.g. pain and hemarthrosis). There is, however, no strict definition for what should be regarded as early and delayed ACL reconstruction, and the timing of ACL reconstruction varies considerably between geographical regions [88]. Surgery within 3 weeks has been defined as an early ACL reconstruction [34, 101], although this definition is not consistent and a recent literature review found that the definition of early ACL reconstruction ranged from 2 days to 7 months among the included trials [2]. For correct interpretation of the pre-treatment assessment, it is important that the time from injury to pre-treatment assessment is always reported, as outcomes may be very different for a patient who is completing such an assessment soon after injury compared to a patient who was injured many years previously.

The impact of the ACL injury on the patient’s overall well-being and quality of life before treatment should also be measured [73, 86]. A Health-Related Quality of Life (HRQoL) measure covers a larger picture of how an ACL injury affects a patient in terms of physical, social and emotional health, which must not be overlooked among patients sustaining an ACL injury [35]. Pre-treatment assessment of HRQoL allows for evaluation of health status over time and whether the treatment restores the patient to better, similar or worse health. Most measures of HRQoL also have the advantage of providing the possibility to determine utilities that are used in estimating the economic impact of the injury and allow for comparison between many other conditions and treatments. A list of HRQoL measures is provided in Table 4.

**Table 4:** Health-related quality of life outcome measures

Instrument	Developer	No. of items	Response options
KOOS [92]	Roos et al.	42 items of which 5 are related to QoL	Each item scored 0–4
ACL-QOL [70]	Mohtadi et al.	32 items	A 100-mm VAS for each item
SF-8 [18]	Quality Metric	8 items	Each item scored on a 6-point scale
EQ-5D [32]	EuroQoL	6 items	Item-specific
SF-36 [112]	Ware and Sherbourne	36 items	Item-specific
SIP [15]	Bergner et al.	136 items	Yes/no
QWB [3]	Anderson et al.	71 items	Via interview

*ACL-QOL* Quality of Life Outcome Measure for Chronic Anterior Cruciate Ligament Deficiency, *EQ-5D* European Quality of Life-5 dimensions, *KOOS* knee injury and Osteoarthritis Outcome Score, *SF-8* short-form-8 health survey, *SF-36* short-form-36 health survey, *SIP* sickness impact profile, *QWB* quality of well-being

*Conclusion* Description of the sample in terms of demographic characteristics, pre-injury activity level and pre-treatment patient-reported outcomes is necessary to interpret the results of treatment and generalizability of the study.

## Clinical outcome assessment

### Minimal length of follow-up when reporting outcomes depends on the outcome being assessed and should optimally include 80% of the entire cohort

(25/25, 100% agreement)

“80% follow-up rate or more is optimal. Follow-up time should reflect the primary outcome, be based upon the purpose of the study and be stated a priori.”

The follow-up time of a study should be defined depending on what is relevant in relation to the primary investigated outcome. In general, outcomes after ACL treatment can be divided into four categories—early adverse events, PROs, ACL failure/recurrent ligament disruption and clinical measures of knee function and structure (Table 5), all of which could be further stratified in specific outcomes necessitating different considerations for follow-up time as exemplified in Table 6.

**Table 5:** The four robust outcome categories after ACL injury treatment

Adverse events
Patient-reported outcome measurements
ACL failure or recurrent ligament disruption
Clinical measures of knee function and structure

*ACL* anterior cruciate ligament

**Table 6:** Examples of outcome measurements and considerations for follow-up time

Outcome category	Example of specific outcome	Comment
Adverse events	Intraoperative complications	Usually less than one-year follow-up required to detect these outcomes. When identifying adverse events, these should be reported as soon as possible, regardless of the minimum time lapsed from treatment start
	Surgery- or device-related complications	
	Infections	
	VTE	
	Re-operation	
PRO	Validated knee-specific outcome scores	Depending on study purpose, population and the specific outcome tool used. Generally, at least 1 year follow up is required to obtain meaningful measures for interpretation of treatment effect, preferably 2 years. However, for the IKDC-SKF and the KOOS, the 1- and 2-year results have been reported equivalent [81, 95]. Patients could be followed over several years to detect changes over time and to compare short-, mid- and long-term results
	Psychological measures	
	HRQoL	
	Activity level	
	Return to sport	
ACL failure and recurrent ligament disruption	Graft rupture/failure	The follow-up time must allow for sufficient time to detect events such as re-rupture and ACL revision. These events tend to occur after the patient returns to knee-strenuous activities, which means that a 2-year follow-up should be a minimum.
	ACL revision	
	Contralateral ACL injury	
Clinical Measures of Knee function and Structure	Strength testing	Largely depending on the specific outcome and the study purpose. However, care should be taken not to draw conclusion about the short-term treatment result until a 2-year follow-up is obtained. Functional performance tests, knee joint laxity and range of motion assessments are preferably performed in multiple follow-ups prior to the 2-year follow-up for changes over time. Osteoarthritis assessment should have at least 5-year follow-up. Concomitant knee joint injuries should be reported whenever identified
	Hop testing	
	Performance testing	
	Knee joint laxity	
	Range of motion	
	Imaging	
	Osteoarthritis	
	Concomitant knee joint injuries	

*ACL* anterior cruciate ligament, *HRQoL* Health-related quality of life, *IKDC* International Knee Documentation Committee, *KOOS* Knee injury and osteoarthritis outcome score, *PRO* patient-reported outcome, *VTE* venous thromboembolism

Evidence provided by previous research as well as clinical experience is the foundation to determine what a relevant follow-up time is. For example, the rates of ACL re-rupture and ACL revision peaks at 1–2 years after an ACL reconstruction and with a return to sport (RTS) [33, 41, 62, 83, 116]. Therefore, a study with a shorter follow-up than this is not relevant if the primary outcome is re-rupture or ACL revision and a study aiming to make conclusions about ACL treatment failure should not have a follow-up time of less than 2 years and should report RTS as a proxy of risk exposure. In contrast, the outcome of septic arthritis or hardware failure can manifest soon after an ACL reconstruction, [99, 111], and a follow-up time of 6 months or less is sufficient to collect data that will represent a true estimation of such outcomes. Thus, it is important that the follow-up time is defined and based upon the study aims and outcomes.

In most studies, especially with the increasing length of follow-up time, a certain degree of patients lost to follow-up is inevitable. Even a small proportion of patients lost to follow-up can lead to considerable study bias [17], although a common opinion is that a drop-out rate of more than 20% is associated with a serious threat to the internal and external validity and power of the study [16, 102]. A study is therefore recommended to optimally include at least an 80% follow-up rate. However, the possibility of drop-out/retention bias should always be considered when patients are lost to follow-up, i.e. is it possible that the patients who completed the follow-up are different from the patients dropping out? Data should be presented such that the drop-out rate is accurately reported. A strict adherence to the use of checklists is encouraged to facilitate complete data reporting, such as the CONSORT statement [97] for randomized controlled trials and the STROBE statement [122] checklist for cohort studies. Clear step-by-step flow-charts are encouraged. Whenever drop-outs are present, the authors are recommended to perform a drop-out sensitivity analysis to enable interpretation of the possible drop-out effects. This should include a comparison of the baseline characteristics of those that completed versus those that did not complete the study.

It should be emphasized that there can be circumstances where an acceptable follow-up rate for a study is determined by weighing the disadvantages of loss to follow-up against certain advantages, e.g. a long-term follow-up or a considerable amount of data in a study. In such cases, a lower threshold for follow-up rate is acceptable. Large registry studies can be used to exemplify this, where the patient response rates to PROs are a challenge [44]. Registries comprise data on a large number of patients and include multiple follow-up occasions, sometimes over more than a decade [44, 104]. Hence, they are important sources for determining the effectiveness of ACL treatment and for providing hypotheses-generating results [105]. Nonetheless, a large drop-out rate increases the importance of a stringent data reporting and a statistical analysis of patients lost to follow-up also needs to be considered.

*Conclusion* Follow-up time should be determined by the purpose of the study and primary outcome, and should be stated a priori. The follow-up rate should optimally exceed 80% and data must be reported so that the possible effects of patients lost to follow-up can be considered.

### Comprehensive assessment after ACL surgery (minimum 2 years) should include adverse events, clinical measures of knee function and structure, PRO, activity level, and recurrent ligament disruption

(25/25, 100% agreement)

“The comprehensive assessment needs to cover both clinical assessment and the patient’s perspective, and should optimally also include a return to sport.”

A comprehensive assessment following ACL reconstruction should aim to provide a complete picture of outcome related to different dimensions of limitations, which involves numerous aspects of knee-related health and function, objective assessment of hard end-points (Table 6), as well as technical aspects of the surgery (graft choice, fixation, tunnel placement, meniscus/cartilage assessment and treatment). A minimum follow-up of 2 years is likely necessary to enable a comprehensive assessment. Multiple follow-ups during the first 2 years could certainly fulfill the purpose of evaluating, for example, the progress such as in the early-, mid-, and end-state of the rehabilitation. However, the final assessment should be withheld until 2 years postoperatively since a substantial number of outcomes require that this time has been given for the ACL reconstruction to completely heal [48, 84, 121, 124], and for the patient to complete rehabilitation and progress to testing the knee in more demanding activities including full participation in sport or activity. A follow-up of 2 years should allow for determining the patient’s capability of a successful RTS [6] and, importantly, it will include a period when patients are participating at high-risk exposure for ACL failures and re-injuries [33, 41, 62, 83, 116]. An optimal 2-year outcome assessment should, therefore, include reporting of the rate and time of RTS. A consensus statement related to assessment and reporting of RTS was similarly reached at the ACL Consensus Meeting Panther Symposium 2019 and is provided in a separate publication [68].

A comprehensive assessment also implies that the contralateral knee should be examined and assessed for each outcome. Outcome tools such as the IKDC Knee Ligament Standard Evaluation Form [46] require a comparison with the contralateral knee for the standardized reporting. The uninjured contralateral knee serves as a reference for the ACL injured knee in terms of the range of motion, laxity and functional performance [118], which helps to account for differences between individuals. It should also be noted that the contralateral limb/leg/knee might also be affected by an ACL injury such as altered kinematics [54, 69] and a decrease in muscle strength [118], which underscores the importance to ensure that the function of the contralateral limb is optimized before allowing the patient to return to knee strenuous activities by assessing it likewise. It is therefore recommended that the standard practice is to assess the contralateral knee and report such data, which ultimately will contribute to increased knowledge of risk factors for a patient sustaining a subsequent contralateral ACL injury.

Failure of ACL reconstruction is a nonspecific term that is commonly used without a stringent definition in the literature. It is therefore recommended that well-defined outcome assessments are used and that the authors if choosing to use the term failure, report an a priori definition of what a failure is in detail. To define failure as reoperation is verifiable and clear, however, it introduces a risk of underestimating the true failure rate. Other examples of definitions for ACL graft failure include recurrent/persistent instability, pathological anterior or rotatory laxity or evidence of graft failure assessed by MRI or arthroscopy. In overall terms, reasons for ACL failure may be classified as traumatic (e.g. re-injury), technical (e.g. surgical errors) and patient-related (e.g. compliance to rehabilitation, recovery of neuromuscular function or generalized hyperlaxity). Technical errors account for a great amount of all graft failures, with femoral tunnel malposition being a common cause [72, 107]. It has also been reported that previous tibial tunnel malposition is a significant predictor for worse 2-year patient-reported outcome after ACL revision [123]. It is therefore recommended that reporting of ACL reconstruction failure is complemented by reporting of details with regard to the surgical technique. A useful tool is the Anatomic ACL Reconstruction Scoring Checklist (AARSC) [108], which enables grading of surgical variables that define ACL tunnel position in an anatomic manner.

*Conclusion* A minimum of 2-year follow-up is necessary for a comprehensive and reliable determination of the outcome. The comprehensive assessment should include outcomes provided by clinical examination, PROs, activity level and verified re-injuries.

### Comprehensive assessment after ACL surgery in the medium to long-term (5 + years) should also include measures of post-traumatic osteoarthritis

(25/25, 100% agreement)

“A common methodology of outcome assessment for osteoarthritis is needed and should be included in mid- to long-term follow-up studies.”

It is well known that sustaining an ACL injury entails a high risk of developing post-traumatic OA in the mid- to long-term, especially if concomitant intra-articular injuries are present [1, 22, 82, 87]. Reducing the risk of OA is a clinical priority, which means that the mid- to long-term follow-up assessment should include measures of OA to monitor and evaluate the degenerative changes in the knee joint. This is necessary for developing therapeutic interventions aiming to counter the high rate of OA after an ACL injury.

Measures of OA may include clinical examination, PROs and imaging modalities. Clinical examination findings that may indicate OA are joint line tenderness or crepitus, which previously have been found to be strong predictors for OA [96]. Good inter-observer reliability for joint line tenderness and crepitus has been reported when a standardized approach is used [66]. The IKDC Knee Ligament Standard Evaluation Form includes a grading system for such an examination and should be used for standardized reporting [46].

The use of PROs is valuable to capture the patients’ perception of impairments caused by OA. Questionnaires specifically developed and validated for assessment of OA are the WOMAC [13] and the KOOS [92]. However, the WOMAC was developed for evaluation of established OA, as such the KOOS may be a more appropriate assessment for patients following ACL injury. This is because the KOOS is more likely to detect early development of OA compared with WOMAC since the KOOS was developed to cover a broader spectrum, from a knee injury to manifest OA [92, 93].

Imaging modalities still provide the most sensitive assessment of OA although not without limitations. One should remember that radiographic findings of OA are not necessarily accompanied by symptomatic OA [4, 85], and other intra-articular pathologies may give similar symptoms as OA. It is therefore recommended to combine radiographic imaging assessment with PROs for decision-making when it comes to symptomatic OA. Radiographic findings should be described in a standardized manner using validated tools, where the Kellgren–Lawrence [56] perhaps is the most commonly used tool, taking into account osteophyte formation, sclerosis, joint space narrowing and bone deformity [56]. Although plain radiography has long been the established method for imaging of OA it must be acknowledged that the modality has a limited capacity to visualize early stages of OA and to grade OA progression [64].

The rapid evolution of MRI techniques enables a much more comprehensive assessment of knee joint structure, such as early morphological and biochemical changes of articular and periarticular structures. Quantitative measurements of cartilage thickness on MRI have a higher sensitivity for knee OA compared with traditional radiological measures [120]. In addition, MRI detects characteristic OA signs, earlier and with a greater sensitivity compared with radiography [42]. Structural intra-articular changes are indicative for OA and can be seen as early as 2 years after an ACL reconstruction with MRI, which is earlier than these changes can be seen on radiographs [20, 109]. In addition, MRI can also rule out other intra-articular injuries that may explain symptoms perceived by patients. Thus, although plain radiography has an established role in the assessment of OA and is favorable from an availability and cost perspective, its main role is to assess the development of OA in the long-term and for already established OA. For early- or mid-term assessment of OA, attempts should be made to include MRI to detect early changes with greater validity and sensitivity [42].

It is not known when clinically relevant post-traumatic OA occurs, or when in this process the structural changes of the knee joint start to appear. With the advancement in imaging techniques there is a risk of over-diagnosis of OA since structural changes without clinical significance might be detected. Future research will hopefully provide a clearer picture of this, as well as methods to distinguish between what are pathological changes and what changes are related to normal aging [65]. Until then, an assessment of knee OA should always be made in relation to a “control knee” to provide a reference for such variables. A synthesis of current literature shows that the contralateral knee is most commonly used for this purpose, followed by using an age- and sex-matched control group [87]. The latter methodology, using a separate comparison group, is the preferred method since degeneration can occur in the contralateral knee although it was not part of the original injury. Some studies have used baseline imaging of the acute ACL injured knee as the control [1], which cannot be recommended since this method does not take into account the impact of natural aging occurring between the injury and the long-term follow-up.

*Conclusion* Outcome assessment of OA should include clinical examination, PROs and imaging modalities, for which MRI is the preferred modality for increased accuracy. Imaging findings should always be set in context with the patient’s perception and the clinical examination for decision-making. Hence, these outcome assessments are equally important for determining the outcome of OA.

### Clinical assessment of ACL injury treatment should include measures of anteroposterior and rotatory knee laxity

(25/25, 100% agreement)

“Evaluation of knee joint laxity is a cornerstone for evaluating the outcome of ACL treatment. Quantitative measures of knee joint laxity increase the reliability and validity”.

The anatomic properties of the ACL make it a primary passive restraint to both anteroposterior (AP) and rotatory forces of the knee joint [47]. Valid assessment of knee joint laxity is therefore key in the evaluation of the outcome of surgical treatment after ACL injury, preferably at multiple follow-ups to detect any changes over time. Failure to eliminate knee joint laxity with ACL reconstruction could indicate treatment failure, while patients undergoing non-operative treatment should be assessed for excessive laxity or propagation of knee joint laxity. The latter scenario might be an indication for subsequent operative treatment, although the term laxity should be distinguished from instability or stability. Knee joint laxity is defined as the passive response of the knee joint when an external force or torque is applied, while instability is the patient’s perception of symptoms during functional movement independent of laxity [79]. Hence, knee joint laxity can be reliably measured and reported, which makes it the preferred metric for clinical outcome assessment. To minimize the risk of bias, every attempt should be made to blind the assessors and all participating assessors should be trained in using a standardized execution technique of the laxity test.

Laxity assessment consists of static and dynamic examinations, and methods for both grading by the examiner and quantification of laxity have been developed. Laxity assessments should always include a side-to-side comparison with the contralateral knee. Static AP knee laxity tests consider a single degree of freedom of motion and includes application of a unidirectional force in a single plane, such as the Lachman test and the anterior drawer test. The IKDC Knee Ligament Standard Evaluation Form provides a standardized classification of the degree of AP translation [46]. For instrumented quantitative assessment of AP laxity, the KT-1000/2000^®^ [28] and the Rolimeter^®^ [8] provide among the most accurate measurements, although the intraclass correlation coefficient (ICC) is variable according to the literature and the results are examiner-dependent [89]. Another instrument is the GNRB^®^ (Genourob, Laval, France), which is a robotic arthrometer developed to alleviate the difficulties with examiner-dependent measurements. The patient’s leg is placed in the robotic system and a pre-defined force is applied to the proximal calf, while the relative displacement of the anterior tibial tubercle with respect to the patella is recorded by a displacement sensor. The GNRB also offers the advantage of using electromyography sensors to record hamstring activity, in order to detect incomplete hamstring relaxation that affect the result [90]. Static AP measurements do not necessarily correlate with clinical outcome and function [7, 58, 59], which indicates that laxity assessment should not solely rely on static AP translation since it fails to capture the more complex knee kinematics.

**Fig. 2:**
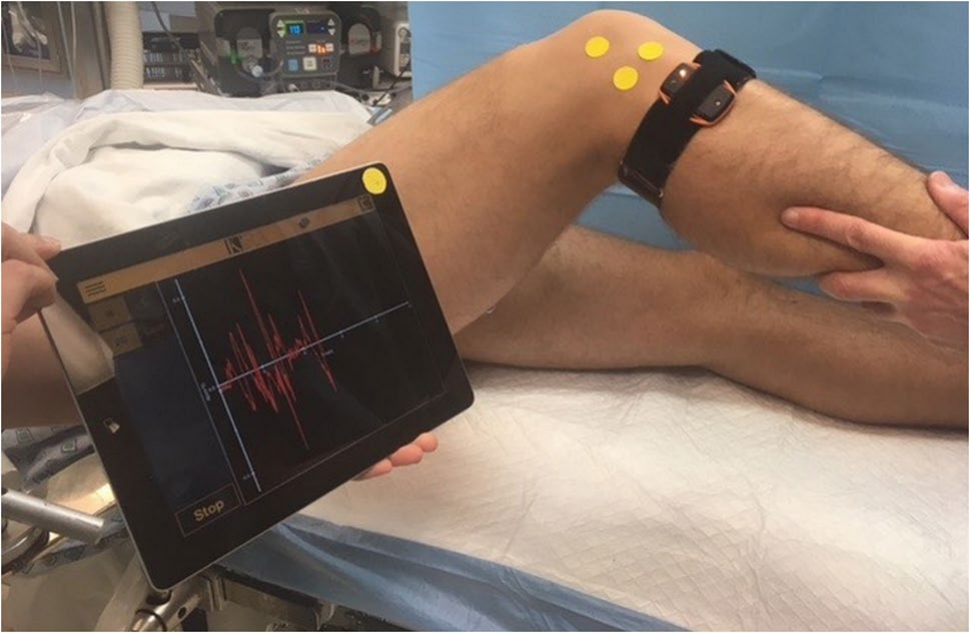
The KiRA inertial sensor system for quantifying lateral tibial acceleration during the pivot shift test

**Fig. 3:**
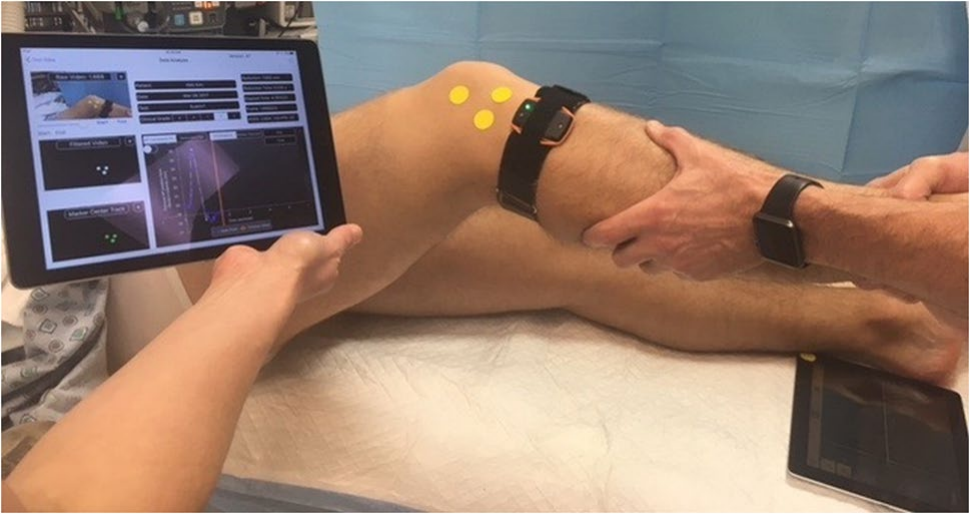
Image analysis system on iPad for quantifying lateral tibial translation during the pivot shift test

The pivot shift (PS) test is considered to simulate a more physiologic multiaxial loading of the knee joint since it is a dynamic test of laxity that evaluates both AP and rotatory laxity [49]. It has been reported as the most specific test for ACL deficiency [14]. On the other hand, the PS is characterized by a large variability in execution techniques [61, 78], which may lead to a variation in clinical grading between examiners. To overcome this, a standardized PS test has been described, which has led to an improved accuracy of the test [78]. Moreover, user-friendly devices for non-invasive quantitative PS have been developed and determined to be valid for objective assessment of the PS [77]. Such devices may include an inertial sensor system (KiRa, Orthokey LLC, USA) [125, 126] to quantify the tibial acceleration during the PS and an image analysis system [75] which enables a quantification of the lateral tibial translation during the PS. Both devices have been shown to be able to validly detect differences between clinically high- and low-grade PS (Figs. 2 and 3) [77]. Example of devices for quantitative AP and rotatory knee laxity that are easily applicable in the clinical setting are summarized in Table 7.

**Table 7:** Devices for quantitative assessment of knee joint laxity

Device	Accuracy	Comments
KT-1000/2000^®^	The majority of studies show at least a fair reproducibility (inter-tester ICC range 0.14–0.92, intra-tester ICC range 0.47–0.95) [89]	Measure anterior tibial displacement in mm.
		Different reliability depending on examiner experience [89]
		Dependent on dominant hand of the examiner [100]
		The maximal manual force testing is the most reliable [89]
Rolimeter^®^	The literature shows an inter-tester correlation ranging between 0.39 and 0.89 and intra-tester ranging between 0.55 and 1.0 [74, 98]	Measure anterior tibial displacement in mm.
		Not as crucial with examiner experience compared with the KT-1000 [74]
		Might be easier to apply in the clinical setting compared with the KT-1000 due to the lighter design. At least as reliable as the KT-1000 [89]
GNRB^®^	Sensitivity and specificity for an ACL tear ranging between 62% to 92% and 76% to 99%, respectively [11, 57, 90]	Measure anterior tibial displacement in mm
	The inter-tester ICC has been reported ranging between 0.220 and 0.424 [114]	Robotic testing meaning a less examiner-dependent measurement. Several studies reporting the GNRB as reliable or superior to other arthrometers [91]
		Possible to account for patient guarding with hamstring activation [90]
Pivot App	Excellent inter- and intra-tester reliability reported. Inter-tester ICC 0.95 (95% CI 0.54–1.00), intra-tester ICC ranging between 0.91 and 0.99 (95% CI 0.319–1.000) [75]	Lateral tibial translation during the pivot shift test is calculated (in mm) by a software program analyzing the movement of three markers placed on the skin during video recording of the pivot shift test using a commercial tablet
		Been proved valid to detect differences between clinically high- and low-grade pivot shift [77]
KiRa	Mean intra-rater ICC 0.79. Reproducibility is good to excellent across all different parameters being quantified (minimum, maximum and range of tibial acceleration) [63]	An inertial sensor system quantifies the tibial acceleration (m/s^2^) during the pivot shift test. An elastic strap is used to position the sensor on the patient’s leg when executing the pivot shift test
		Has been proved to be valid to detect differences between clinically high- and low-grade pivot shift [77]

*ACL* anterior cruciate ligament, *ICC* intraclass correlation coefficient

*Conclusion* Knee joint laxity should be assessed after ACL treatment and reported in a standardized manner using the IKDC Knee Ligament Standard Evaluation Form when clinical grading is used. The use of quantitative measures is encouraged to increase the reliability and validity of the assessment.

## Patient-reported outcome

### Assessment of patient-reported outcome (PRO) should optimally include at least one knee-specific outcome tool, one activity rating scale, and one measure of health-related quality of life

(25/25, 100% agreement)

“There is a fine balance between multiple outcome assessments and the responder burden in clinical outcome assessment”

The use of PROs has become a cornerstone for researchers to understand the patients’ perspective on the impact of ACL injury and treatment. During recent decades, technical development has facilitated the use of PROs as patients can report and researcher can collect responses electronically. The time-efficient collection has tempted researchers to burden patients with more PROs in studies. Responder burden is an important term in research and is defined as the time to complete items as well as the physical energy and cognitive demands placed on those responding. In addition, all clinical testing of patients is part of the burden placed on our patients. Because of the risk of excessive responder burden, which threatens the validity of an individual’s responses and thus their score, researchers are advised to wisely choose PROs specific for the study purpose.

Similar to statement number 2 of this consensus paper on baseline information to collect, it is recommended to use at least one knee-specific tool, one HRQoL tool and one activity rating scale. This provides the researcher with a comprehensive picture of the patients’ perception of outcome after treatment.

*Conclusion* To give a comprehensive assessment of the patients’ perception of the impact of ACL injury and outcome of treatment, validated knee-specific PRO assessment, HRQoL and measure of type and level of pre-injury sport/activity should be collected before and after treatment.

### The IKDC Subjective Knee Form is the recommended knee-related outcome measure for ACL injury and treatment

(24/25, 96% agreement)

“It is important to find a universal metric—the IKDC-SKF is currently the optimal scale, but we should be careful not to neglect the other scores”

The evaluation of treatment outcome started historically with the use of objective measurements as proxies for what clinicians and patient really cared about. For instance, both rating scales and measures of ROM, strength and laxity were frequently used, however, these measures are limited by inter- and intra-rater variability and alone failed to determine symptoms and limitations perceived important by the patient. Failure to report and quantify the patients’ perspective of treatment outcome after ACL injury led to the development of knee-related PROs during the late 1990’s and early 2000’s. The two most commonly used PROs after ACL injury are the KOOS and the IKDC-SKF, which were both developed during this time period. Measurement properties of the IKDC-SKF and KOOS are presented in Table 8.

**Table 8:** Psychometric properties of the IKDC-SKF and the KOOS [38]

	IKDC-SKF	KOOS
PASS	75.9	Pain = 88.9
		Symptoms = 57.1
		ADL = 100
		Sport = 75.0
		QoL = 62.5
MCID	11.5	N/A
MIC	10.9	Pain = 2.5
		Symptoms = -1.2
		ADL = 2.4
		Sport = 12.1
		QoL = 18.3
MDC	11.5	Pain = 6.0–6.1
		Symptoms = 5.0–8.5
		ADL = 7.0–8.0
		Sport = 5.8–12.0
		QoL = 7.0–7.2
Content validity	Poor	No evidence
Structural validity	No evidence	No evidence
Internal consistency	0.77 to 0.97	Pain = 0.84–0.91
		Symptoms = 0.25–0.75
		ADL = 0.94–0.96
		Sport = 0.85–0.89
		QoL = 0.64–0.9
Measurement error	3.2 to 5.6	Pain = 2.2–10.1
		Symptoms = 3.1–9.0
		ADL = 2.9–11.7
		Sport = 2.1–24.6
		QoL = 2.6–10.8
Test Re-Test Reliability	0.85 to 0.99	Pain = 0.85–0.93
		Symptoms = 0.83–0.95
		ADL = 0.75–0.91
		Sport = 0.61–0.89
		QoL = 0.83–0.95
Responsiveness	Good	Poor
Cross-cultural validity	Fair	No evidence

*ADL* activities of daily living, *MCID* minimal clinically important difference, *MDC* minimum detectable change, *MIC* minimally important change, *PASS* patient acceptable symptom state, *QoL* quality of life

These PROs have advantages and disadvantages, and when choosing between them, one should evaluate what the population is and what it is that you want to capture. Most importantly, measurements should consist of those that are relevant to the patient and capture the full range of symptoms, activity limitations and participation restrictions to increase the relevance and validity in results attained from PROs [24]. It is essential that the PROs have undergone rigorous validation to the target condition to be able to differentiate better from worse treatment outcome. The inappropriate use of a PROs can distort results from a study and cause difficulties to detect differences as items may not be relevant for the given population. This can be the case when a questionnaire aimed to assess outcome in patients with OA is used to assess patients with an ACL injury.

The KOOS is an extension of the WOMAC [12] (covers the subscales of pain, symptom and limitations in ADL) and was validated for patients with OA of the knee. The initial idea of the KOOS was to develop a region-specific outcome to capture the progression of knee-related symptoms across the lifespan of a patient, from a knee injury to the development of OA. Despite the inclusion of the sport and recreation and quality of life subscales, the KOOS has limited measurement properties in the three original WOMAC subscales when used for patients after ACL reconstruction [24, 60]. It is also worth mentioning that the hybrid version of the KOOS, the KOOS_4_ (a modified version where the items related to activities in daily living have been excluded to avoid ceiling effects) [37], has not undergone a validation [23, 24]. This is problematic as the ability to detect differences between treatments will be limited with the KOOS used in patients with an ACL injury [60]. Using patient-reported outcome measurements that include items that are not relevant or do not cover important limitations of the target condition is not optimal. Using such PROs entails a potential wash-out of treatment effects, inadequate measurement properties and risk of false-negative findings [24, 95, 103]. In terms of the KOOS, several questions are at risk for a ceiling effect when used in patients after ACL reconstruction, i.e. the item is too “easy” for the patient. In addition, the KOOS does not include specific items relating to instability, which is one of the most common symptoms and one of the strongest indications for an ACL reconstruction. The KOOS consists of 42 items entailing higher responder burden compared with other outcomes such as the IKDC-SKF. Awareness of the limitations of the KOOS for the patients after an ACL injury or reconstruction is important to avoid missing the effects of treatment results.

The IKDC-SKF was developed as a region-specific outcome relevant for a variety of conditions including ligament and intra-articular pathologies [51]. This PRO underwent rigorous testing during its development including a reduction from 42 to 18 items and an exploratory factor analysis suggesting that it was reasonable to combine the items into a single overall score. To test the relevance of the IKDC-SKF for patients with an ACL injury, Rasch-analysis was performed separately for patients with and without knee ligament injury [51, 110]. The analysis supported the premise that the items of the IKDC performed similarly in terms of difficulty for individuals with or without a ligament injury. The results from the primary testing of the IKDC-SKF also indicated that the IKDC-SKF items performed the same regardless of age, sex and a variety of diagnoses including ligament, meniscal, articular cartilage injury and patellofemoral pain [30, 51].

The IKDC-SKF is recommended as the knee-related PRO to use for patients after ACL reconstruction because of its quick-to-use 18 items [51]. The IKDC-SKF shows adequate internal consistency and has no floor or ceiling effects across mixed groups of patients with knee conditions [30]. It also has high levels of test re-test reliability, construct validity, and responsiveness. Moreover, normative data has been determined, which is valuable for comparisons, as well as cut-offs for what the patients consider as an acceptable symptom state [53].

There are also other promising PROs used to cover different aspects of recovery after ACL reconstruction, including the Quality of Life Outcome Measure for Chronic Anterior Cruciate Ligament Deficiency (ACL-QoL) [70] and the Knee Numeric-Entity Evaluation Score (KNEES-ACL) [25]. The ACL-QoL is used to determine the effectiveness of ACL reconstruction or any other treatment, and is a 32-item condition-specific quality-of-life scale for patients with ACL deficiency [70]. The KNEES-ACL was developed in 2013 [25], and the thorough development process and dimensionality assessment resulted in 42 items across 7 latent constructs. There is strong positive evidence given to content validity [25, 26].

The ACL-QoL and the KNEES-ACL are promising outcome measurements and likely will help us to better understand patients who have sustained an ACL injury. However, these PROs have mainly been used in comparative studies and are yet to be compared with the established IKDC-SKF and KOOS to prove their respective strengths of constructs.

*Conclusion* The IKDC Subjective Knee Form (IKDC-SKF) is the recommended knee-related outcome measure for ACL injury and treatment.

### Measurement of the patient acceptable symptom state (PASS) is valuable in the assessment of the outcome of ACL injury and treatment

(25/25, 100% agreement)

“One question can carry the advantage of giving the patient the opportunity to tell the story.”

As researchers and clinicians of today, we are equipped with a great variety of PROs. However, the development and use of these PROs means little if the results are not interpreted in a clinically meaningful manner. The use of numeric scores poses a risk that researchers focus myopically at numbers and statistically significant findings, without reflecting over whether such findings really are impactful from the patient’s perspective. For many such PROs, the same score can be achieved despite that patients respond differently to the items that comprise the PRO measure. The question of whether the patient perceives an acceptable symptom state is a priority for all clinicians and the use of the patient acceptable symptom state (PASS) in PRO assessment is important. The PASS considers a single-item question and aims to determine a threshold beyond which the patients consider themselves ‘well’ [76]. Thresholds for the PASS have been established for the KOOS and the IKDC-SKF by asking the question: “Taking account of all the activity you have during your daily life, your level of pain and also your activity limitations and participations restrictions, do you consider the current state of your knee satisfactory?” alongside the administered PRO [76]. Several studies have since then applied the PASS values for the KOOS and IKDC-SKF when reporting on outcome after ACL treatment [27, 43, 45, 117].

A single-item outcome like the PASS summarizes the patient’s perception and allows the patient to make an overall statement through a binary answer, ‘yes’ or ‘no’. A numeric scale might have its advantages; however, it is associated with difficulties of interpretation for both patients and researchers. That is, what is considered as a good and poor outcome, respectively? The PASS reference value at which a majority of the patients feel well is valuable for determining this important question, and its use is warranted to overcome limitations with numeric PROs such as ceiling effects and poor responsiveness [50, 73].

In addition, the evidence to support the interpretation and use of a PROs should include the minimum detectable change (MDC) score and the minimal clinically important difference (MCID) score. These scores collectively describe the responsiveness of the PRO, which is the ability to detect a clinically important change in outcome for the metric. The MDC is the amount of change that is needed to confidently state that the change is beyond measurement error [10]. Thus, if a study finds a difference that is smaller than the MDC for the chosen PRO, one should be careful to draw any conclusions since the observed difference is within the range of measurement error for the PRO. On the other hand, if the change in outcome is larger than the MDC it still remains unknown whether this change is clinically relevant. This is where the MCID becomes valuable. If a change in outcome exceeds the value of the MCID for the PRO, the difference is likely to be perceived as important by most patients [52].

*Conclusion* The PASS is a valuable complement to numeric PROs and should be used to facilitate interpretation of PROs. Researchers should also consider the MDC and MCID for the PRO when reporting and discussing their study findings.

## Future directions

Reaching consensus for clinical outcome assessment after ACL treatment is an important step towards refining and improving the quality of ACL research. Further efforts should be made to develop methods for outcome assessment that provide the most relevant and valid data for patients receiving ACL treatment. A focus is to improve the PRO assessment. The collection of PROs has become increasingly important among health-care professions. Not only is it a valuable asset for a clinician to understand a patient’s perception of health and results of treatment it has also gained importance for policy-makers in determining healthcare quality and developing a value-based healthcare [73]. Commonly used PROs in ACL research are limited by a format of fixed-length surveys that many times include items of questionable relevance for the young and active population sustaining ACL injuries, leading to ceiling effects and potentially survey-fatigue. Therefore, a current priority is to decrease the responer-burden for patients in PRO assessment.

Improved PRO data collection may be achieved through the use of the item response theory (IRT) [21, 36], which has enabled the introduction of computer adaptive testing (CAT). The underlying premise of IRT is that the way an individual responds to an item (question) is based on the difficulty of the question and the ability of the individual. When administered as a CAT, a mathematical algorithm is utilized to select items that are matched to the ability of the patient. For example, if an individual responds to an item that he/she is unable to walk a mile, the computer algorithm will bypass “harder” items such as running a mile and select an easier item such as the ability to walk a block. This means that only items that are relevant about the individual’s ability level are administered, which substantially reduces the time and burden associated with administration of PROs. Efforts are underway to convert the IKDC-SKF to a CAT format that is based on IRT.

Although computer-aided PRO assessment likely is the future, further research for optimization of currently used PROs is needed. Research should focus on determining the most responsive items of current PROs to condense the surveys to include only the most responsive questions. This is important when considering the already collected PRO data for tens of thousands of patients in large registries and national databases. Such data might need to be re-analyzed using the condensed PROs and thereby provide results with a greater precision on clinically relevant outcomes.

Other important aspects for further research is outcome measures on activity and RTS after ACL treatment. Optimally, a tool that is able to quantify sports participation in terms of level, volume and intensity should be developed and implemented as a standardized tool used across studies. With the rapid evolvement of technology, the future will likely also hold easily accessible use of quantitative instruments for quantitatively measuring patient activity. For example, the use of GPS and motion detectors during sports participation, measurements of joint function and measurements of heart rate and speed to estimate intensity.

## Conclusion

Clinical outcome assessment after ACL injury can be divided into four robust categories—early adverse events, PROs, ACL failure/recurrent ligament disruption and clinical measures of knee function and structure. A minimum of 2-year follow-up is necessary for a comprehensive and reliable determination of outcome, which should include outcomes provided by clinical examination, PROs and verified re-injuries. The PRO assessment is a cornerstone in evaluating outcome after ACL injury, where validated knee-specific PRO assessment, HRQoL and measure of type and level of sport/activity should be collected. The IKDC-SKF is the recommended knee-related PRO measure for ACL treatment and the use of PASS is encouraged to facilitate interpretation of PROs.

## Electronic supplementary material

Below is the link to the electronic supplementary material.

Supplementary material 1 (DOCX 16 kb)
