# Supplementary material for: Clinical outcomes after anterior cruciate ligament injury: panther symposium ACL injury clinical outcomes consensus group
Source: Knee Surg Sports Traumatol Arthrosc. 2020 Aug 6;28(8):2415–34. doi: 10.1007/s00167-020-06061-x (PMC7429530; doi:10.1007/s00167-020-06061-x)
Supplement: Supplementary file 1 — Supplementary material 1 (DOCX 16 kb) [file 167_2020_6061_MOESM1_ESM.docx]

Panther Consensus 2 Outcomes – Initial survey data

Answer choices: strongly agree, somewhat agree, neither agree nor disagree, somewhat disagree, strongly disagree

Q1 - Short-term outcome assessment should include a minimum 2 years and 80% follow up of the entire cohort.

78% strongly agree

17% somewhat agree

5% somewhat disagree

Q2 - Medium-term outcome assessment should include a minimum 5 years and 80% follow up of the entire cohort.

70% strongly agree

30% somewhat agree

Q3 - Assessment of patient reported outcome (PRO) should include one knee-specific outcome tool, one activity rating scale, and one measure of health-related quality of life.

50% strongly agree

50% somewhat agree

Q4 - The IKDC Subjective Knee Form is the accepted knee-related outcome measure for ACL injury and surgery.

61% strongly agree

22% somewhat agree

13% neither agree nor disagree

4% strongly disagree

Q5 - A single numerical measure is sufficient for assessing overall outcome following ACL injury/surgery.

13% strongly agree

8% somewhat agree

29% neither agree nor disagree

25% somewhat disagree

25% strongly disagree

Q6 - The evidence to support the interpretation and use of a PRO for the knee should include:

- The minimum detectable change (MDC) score
- The minimal clinically important difference (MCID) score
- The patient acceptable symptom state (PASS) score
- Normative data from a large and representative sample

52% strongly agree

35% somewhat agree

13% neither agree nor disagree

Q7 - After ACL surgery, short-term outcome assessment (2+ years) should include PRO, return to play, and graft failure/re-injury.

82% strongly agree

18% somewhat agree

Q8 - After ACL surgery, medium to long-term outcome assessment (5+ years) should include OA progression, PRO, and graft failure/re-injury.

64% strongly agree

36% somewhat agree

Q9 - Rotatory knee laxity outcome should be assessed as an outcome following ACLR.

33% strongly agree

54% somewhat agree

8% neither agree nor disagree

4% somewhat disagree

Q10 - For acute injury, final functional status is the outcome of interest, and minimum pre-operative outcome reporting must include demographic information and recording of type and frequency of pre-injury activity and participation.

50% strongly agree

46% somewhat agree

4% strongly disagree

Q11 - For chronic injury, improvement from pre-surgery status is the outcome of interest, and minimum pre-operative outcome reporting must include full PRO assessment (demographic, knee-specific, activity-level, HRQoL).

48% strongly agree

52% somewhat agree

Q12 - For medium to long-term outcome assessment (5+ years), OA progression is the outcome of interest, and baseline radiographic status of the ipsilateral knee and concurrent radiographic status of the contralateral knee must be reported.

38% strongly agree

42% somewhat agree

4% neither agree nor disagree

13% somewhat disagree

4% strongly disagree

Q13 - Sample sizes of 400 to 500 are required for studies assessing absolute re-injury risk reduction of 10% in high-risk patients.

25% strongly agree

42% somewhat agree

29% neither agree nor disagree

4% somewhat disagree
